# Organic electrochemical transistors based on a dielectrophoretically aligned nanowire array

**DOI:** 10.1186/1556-276X-6-339

**Published:** 2011-04-14

**Authors:** WooSeok Choi, Taechang An, Geunbae Lim

**Affiliations:** 1 Department of Mechanical Engineering, POSTECH, 790-784 Pohang, Republic of Korea; 2 Division of Integrative Bioscience and Biotechnology, POSTECH, 790-784 Pohang, Republic of Korea

## Abstract

In this study, we synthesized an organic electrochemical transistor (OECT) using dielectrophoresis of a carbon nanotube-Nafion (CNT-Nafion) suspension. Dielectrophoretically aligned nanowires formed a one-dimensional submicron bundle between triangular electrodes. The CNT-Nafion composite nanowire bundles showed p-type semiconductor characteristics. The drain-source current decreased with increasing gate voltage. The nanowire bundles showed potential as pH sensor because the drain-source current ratio varied linearly according to the gate voltage in pH buffers.

## Background

Recently, there has been significant research in the area of organic thin-film transistors (OTFTs), because of the many benefits of organic semiconductors, such as structural flexibility, low temperature processing, and low cost [1-7]. Organic electrochemical transistors (OECTs), a subset of OTFTs, have been considered as sensors because of their ability to operate in aqueous environments with relatively low voltages and their integration with microfluidics. Furthermore, one can to get information on additional dimensions using gate-induced modulation, compared with two-terminal devices [5-12]. In particular, OECTs, formed using one-dimensional nanostructures, such as nanotubes and nanowires, are more attractive for use as chemical and biological sensors because of their large surface-to-volume ratio, light weight, and controllable transport properties [10-13].

**Figure 1:**
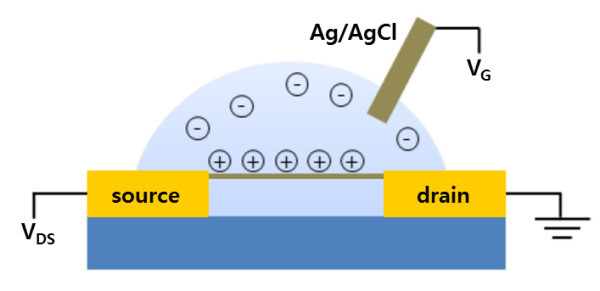
**Schematic diagram of an organic electrochemical transistor based on a CNT-Nafion nanowire bundle**.

Recently, we have developed a real-time, label-free, step-wise, and target-specific aptasensor for protein molecules using dielectrophoretically aligned single-walled carbon nanotube (SWNT) films between patterned cantilever electrodes. We used the SWNT film as a two-terminal resistive sensor and demonstrated its excellent performance for detecting thrombin and vascular endothelial growth factor (VEGF). We verified that the SWNT film had *p*-type semiconductor properties in a phosphate buffer solution at pH 5.6 using blank electrodes of the cantilever array as gate electrodes [14]. The structure of this device can be adapted for OECTs composed of semiconducting material between two electrodes and a remote gate electrode in the surrounding electrolyte solutions (Figure 1) [10-12]. This fabrication method is applicable to other materials under positive dielectrophoretic conditions. In addition, CNTs offer mechanical support to the organic materials, and their composites can improve electrical properties, such as conductivity, conductance, and electronic transport [15-20]. Our objective was to synthesize CNT composite nanowires aligned between electrodes using dielectrophoresis and to exploit them as OECTs for sensor applications.

In this article, we report the fabrication of CNT composite nanowires with Nafion, a well-known proton conductor [21,22] and the use of CNT-Nafion composite nanowires as electrochemical transistors in various pH buffers.

## Results and discussion

**Figure 2:**
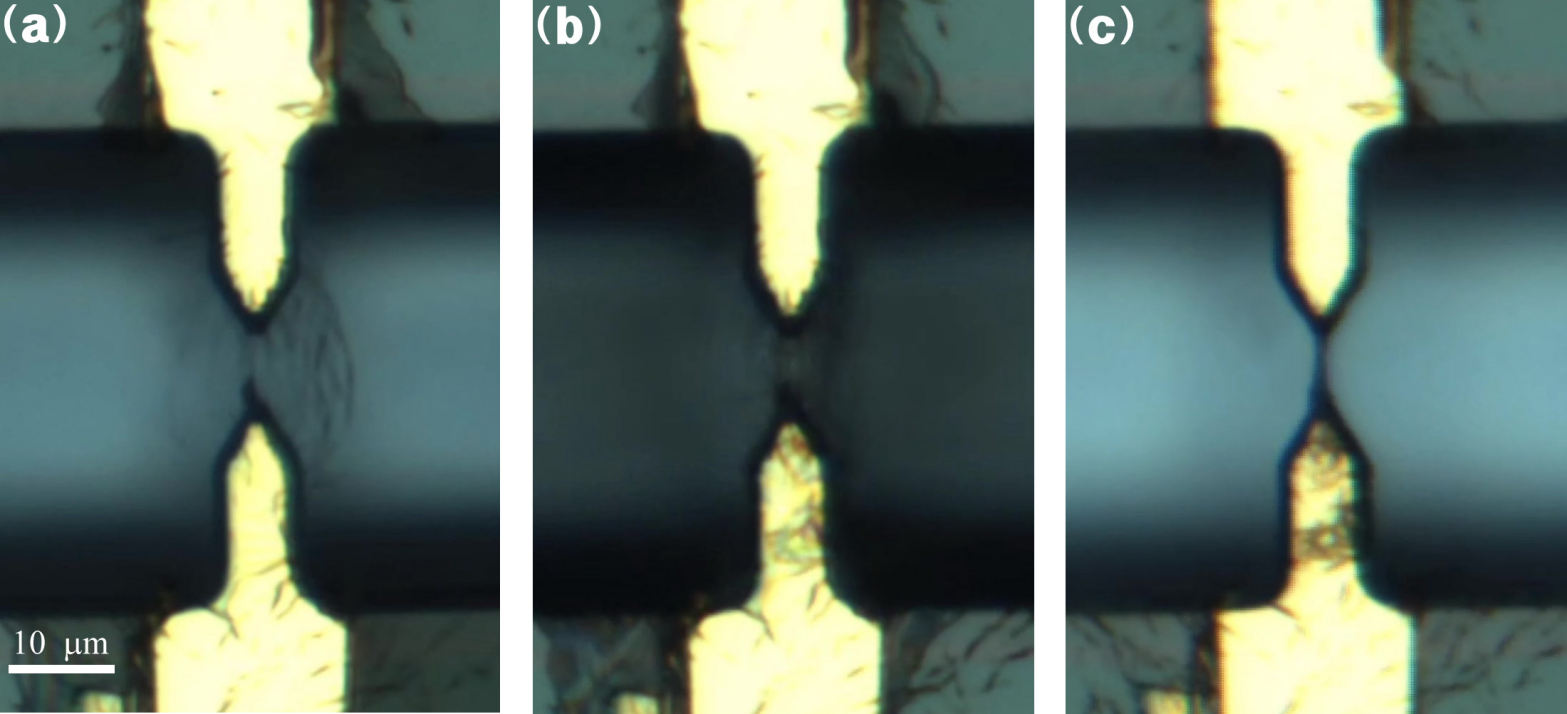
**Microscope images of the CNT/Nafion nanowire fabrication process**. **(a)** Attraction of the CNT and Nafion molecules between electrodes with an AC electric field; **(b)** compression of the CNT and Nafion by suspension evaporation; **(c)** A CNT-Nafion composite nanowire synthesized between electrodes.

Figure 2 shows the CNT-Nafion nanowire synthesis using dielectrophoresis. CNTs and Nafion molecules were gathered between the electrodes where the electric-field gradient was larger, because of their higher conductivity compared with the surrounding medium (Figure 2a). After the suspension was partially removed, the remaining suspension was compressed to form a concave meniscus with evaporation due to the surface tension between the electrodes and suspension (Figure 2b). As a result, the electric current was concentrated through the compressed CNTs and the surrounding Nafion, which bonded the CNT in the shape of the solution. A nanowire bundle with a submicron diameter was synthesized (Figure 2c).

**Figure 3:**
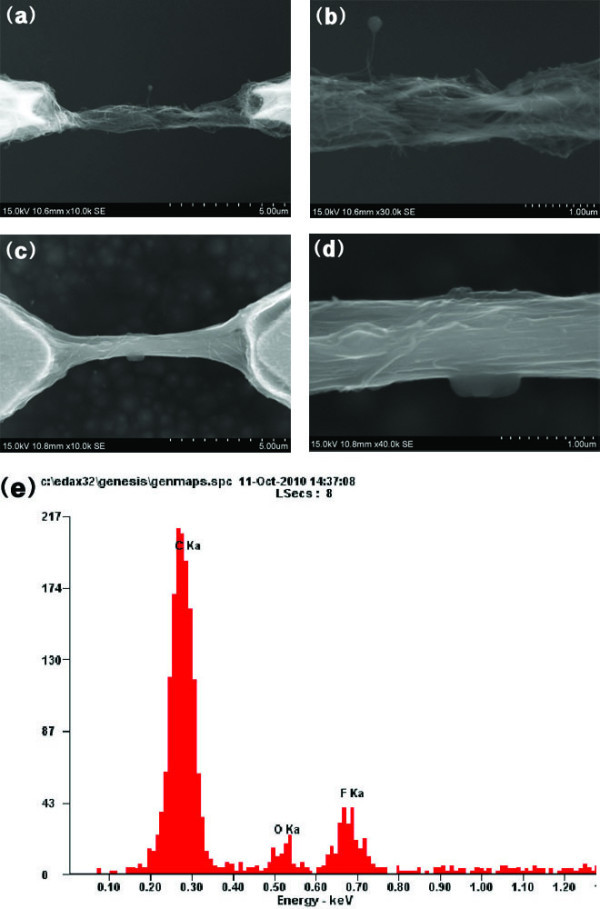
**Difference of CNT and CNT-Nafion composite nanowire bundles**. SEM image of **(a, b)** CNT nanowire bundles and** (c, d)** CNT-Nafion composite nanowire bundles. **(e)** EDS analysis of the CNT-Nafion nanowire bundles.

Figure 3a, b shows a scanning electron microscope (SEM) image of a CNT bundle, and Figure 3c, d shows Nafion-coated CNT bundles. The Nafion wrapped the CNT bundle entirely, while CNT gathered individually. Figure 3e shows the energy dispersive X-ray spectroscopy (EDS) graph of CNT-Nafion nanowire bundles, which were 10% fluorine due to the Nafion composition. Immediately after synthesizing the nanowire bundles, the resistance of the CNT bundles was approximately 5 kΩ. In contrast, that of the CNT-Nafion bundles was found to be approximately 2 kΩ. Based on the SEM image, EDS graph, and electrical properties, the nanowire bundles synthesized were likely CNT-Nafion composites. As we reported previously [14], the SWNT-film was synthesized uniformly between flat cantilever electrodes; however, CNT-Nafion nanowires were synthesized between triangular electrodes. Because the electric field was concentrated at the end of the electrode, and a thin concave meniscus formed during evaporation, the nanowire bundles had submicron diameters, rather than a film structure. This fabrication technique is based on the bottum-up method; consequently, it is a simple method for fabricating CNT nanowire composites using dielectrophoresis.

**Figure 4:**
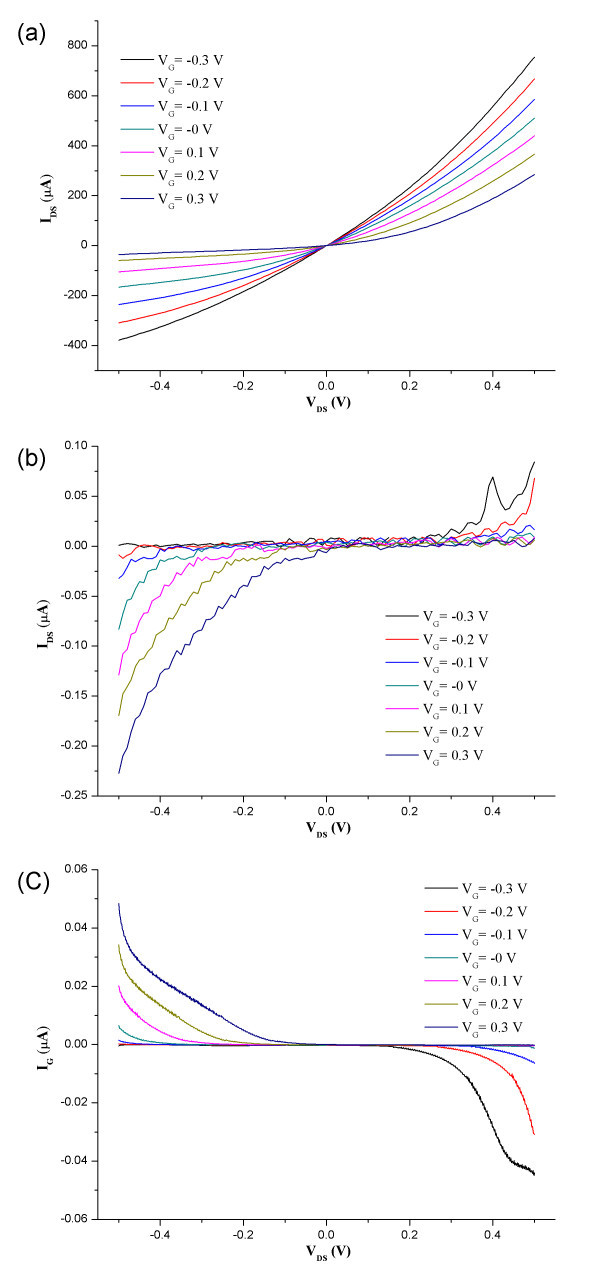
**Verification of CNT-Nafion nanowire electrochemical transistors.** Characteristic curves of I_DS_ versus V_DS_ for **(a)** electrochemical transistors based on dielectrophoretically-aligned CNT-Nafion nanowire bundles and **(b)** blank electrodes in 1 × PBS buffer (pH 7.2). **(c)** Characteristic curves of *I*_G_ versus V_DS_ for the electrochemical transistors under the same conditions.

Figure 4a, b shows the characteristic drain current (*I*_DS_) versus drain voltage (*V*_DS_) curves at different gate voltages (*V*_G_) in 5 μL of a phosphate-buffered saline (PBS) droplet for CNT-Nafion nanowires and blank electrodes, respectively. Figure 4c plots the gate current (*I*_G_) versus *V*_DS _for CNT-Nafion nanowires under the same conditions. The maximum value of *I*_DS _for the nanowire transistor was approximately 700 μA at *V*_G _= 0.5 V. The leakage current, *I*_DS _at the blank electrodes and *I*_G _were at the most 0.2 μA. The leakage current through the electrolyte was negligible because the *I*_DS _value at the blank electrode and *I*_G _were approximately one thousand times smaller than the current through the CNT-Nafion nanowires. The value of *I*_DS _decreased with increasing electrolyte gate bias (Figure 4a), indicating that the holes were the primary charge-carriers in the CNT-Nafion composite nanowires. That is, they exhibited p-type characteristics in the buffer solutions [12,23]

**Figure 5:**
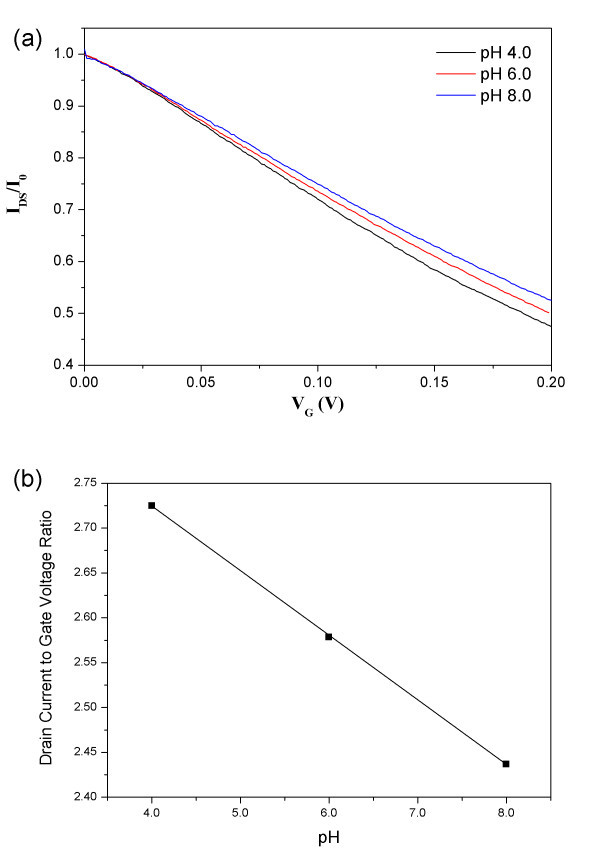
**Characteristics of CNT-Nafion nanowire electrochemical transistors due to pH**. (a) Normalized *I*_DS _versus *V*_G _characteristc curves in various pH buffers when *V*_DS _= 0.5 V. (b) Ratio of the normalized drain current to the gate voltage plotted against the pH of the CNT-Nafion nanowire electrochemical transistors.

To investigate the influence of protons on the characteristics of CNT-Nafion composites, we measured the drain current with increasing gate voltage from 0 to 0.2 V while *V*_DS _was fixed at 0.5 V in various pH buffers. Figure 5a shows the normalized *I*_DS _divided by the drain-source current when *V*_G _= 0 V versus gate voltage characteristic curves in different pH buffers. As expected, because holes were the primary charge-carriers, the normalized drain-current decreased steeperly with increasing gate voltage under high proton concentrations (lower pH). The normalized drain current to gate voltage ratio was linearly dependent on the buffer pH (Figure 5b).

## Conclusions

We fabricated organic chemical transistors based on CNT-Nafion composite nanowires using dielectrophoresis. These composite nanowires had *p*-type semiconductor characteristics in aqueous media, and the drain-current to gate voltage ratio was proportional to the buffer pH. Because the synthesis of nanowire bundles occurred at electrodes with an applied electric field, and various organic materials have the potential to form composites with CNT, one can synthesize an individually addressable CNT composite nanowire array.

## Methods

CNT-Nafion nanowires were synthesized between cantilever electrodes that were fabricated using a traditional MEMS technique. These electrodes were fabricated using a standard lift-off process. A gold layer (2000 Å) was deposited with a chrome layer (200 Å) as an adhesion layer using an e-beam evaporator on a silicon substrate covered with 1 μm of low-stress silicon nitride using low-pressure chemical vapor deposition (LPCVD). For the cantilever structure, the silicon nitride was etched using standard reactive ion etching (RIE), and the silicon was etched using isotropic wet etching using RSE-200 etchant. The SWNTs with 1.0-1.2 nm diameters and lengths of 5-20 μm were purchased from Ilgin Nanotech, and a SWNT-COOH suspension was prepared by oxidizing the CNTs in a strong acid with sonication [24]. Nafion was purchased from Aldrich and was used without purification. The CNT-Nafion solutions were prepared by combining 3 μL Nafion solution and 200 μL CNT-COOH suspension with sonication for 10 min.

The CNT-Nafion solution was placed on the cantilever electrodes, and an AC voltage of 1 MHz and 10 V peak-to-peak was applied. The SWNTs and monomers were aligned between the cantilever electrodes by the dielectrophoretic force. The SWNT-Nafion solution was removed partially while maintaining the AC electric field and the SWNT-Nafion nanowire bundles were synthesized as the remaining solution evaporated.

Figure 1 shows a schematic of the electrochemical transistors, which consisted of two Au electrodes connected by CNT-Nafion nanowires and a remote Ag/AgCl gate electrode immersed in an electrolyte droplet. The electrochemical transistors were characterized in pH buffers using Samchun Chemical at room temperature using a semiconductor analyzer (HP4156A, Hewlett-Packard).

## Abbreviations

CNT-Nafion: carbon nanotube-Nafion; EDS: energy dispersive X-ray spectroscopy; LPCVD: low-pressure chemical vapor deposition; OECT: organic electrochemical transistor; OTFTs: organic thin film transistors; PBS: phosphate-buffered saline; RIE: reactive ion etching; SEM: scanning electron microscope; SWNT: single-walled carbon nanotube; VEGF: vascular endothelial growth factor.

## Competing interests

The authors declare that they have no competing interests.

## Authors' contributions

WSC and GL conceived of the study, and participated in its design and coordination. WSC and TA carried out the experiments. WSC drafted the manuscript. All authors read and approved the final manuscript.
